# CXCL14: the Swiss army knife chemokine

**DOI:** 10.18632/oncotarget.6040

**Published:** 2015-10-08

**Authors:** Charaf Benarafa, Marlene Wolf

**Affiliations:** Theodor Kocher Institute, University of Bern, CH-3012 Bern, Switzerland

**Keywords:** antimicrobial peptide, chemokine, perinatal mortality, mouse model, Immunology

Since their discovery in the late 1980s, chemokines have been hailed as major contributors in the recruitment and function of immune cells in health and in all types of inflammatory diseases. Expression and activity of chemokines and their receptors in cancer cells, stromal cells and tumor-associated myeloid cells have also increased the interest of targeting these molecules in cancer therapy. In addition, direct antimicrobial activity has been reported for many chemokines in vitro but in vivo validation remains scarce. Among the 45 or so members of this family, CXCL14, also named BRAK for its initial isolation from breast and kidney, has a particular status because it is an orphan chemokine for which no receptor has been unequivocally identified. Nevertheless, several reports indicate a chemotactic function for CXCL14 in homeostatic migration of dendritic cells and NK cells. The function of CXCL14 in cancer may depend on the tumor type as both suppressing and promoting activities have been reported. We initially established that CXCL14 had direct killing activity against skin Gram-positive bacteria and *Candida albicans* as well as on Gram-negative gut microbes such as *E. coli* [1].

In a recent study that we wish to highlight here, we have shown evidence that CXCL14 contributes to killing lung bacterial pathogens both in vivo and in vitro [2]. Structurally, CXCL14 has the conserved chemokine fold defined by a three-stranded β-sheet overlaid by a α-helix and maintained by two disulfide bridges. CXCL14 homologs in all vertebrates differ from other chemokines by an abnormally short amino-terminus of only two amino acids located before the first disulfide bridge and normally required for triggering G protein-coupled receptors. We found that the antimicrobial activity of full-length CXCL14 against Gram-negative bacteria was reproduced by N-terminal peptides, particularly by the synthetically produced peptide consisting of the first 13 amino acids as well as by the 17 amino-acid N-terminal peptide generated by proteinase-3, a major neutrophil granule serine protease of neutrophils. Disulfide bonds were not required for this function. The core 14-54 amino acids lacking the N- and C-terminal tails of CXCL14 carried both antimicrobial and chemotactic activities. The C-terminal alpha-helix was dispensable for these functions. Importantly, we demonstrated that CXCL14-deficient (Cxcl14-/-) mice had a defective bacterial clearance in the lungs following intranasal inoculation with Streptococcus pneumoniae. In contrast, the high antimicrobial activity for CXCL14 noted in vitro against *Pseudomonas aeruginosa* did not translate in an essential function in vivo as clearance was unaltered in Cxcl14-/- mice. This finding may be explained by the presence of additional antimicrobial peptides secreted at mucosal surfaces and/or by other cellular mechanisms with overlapping activity against this pathogen. In addition, it remains possible that the antimicrobial activity of CXCL14 in vivo depends in part on activity through a receptor. However, this possibility is unlikely because CXCL14 expression is poorly responsive to inflammatory reactions. In contrast to most chemokines recruiting inflammatory cells, CXCL14 expression is only moderately increased (in lungs [2]) or even downregulated (in skin [1]) following injury or infection.

The elephant in the room regarding the physiological function of CXCL14 remains the cause for susceptibility to premature death of *Cxcl14*^−/−^ mice. According to the MGI database, at least six successful targeting attempts at knocking out the *Cxcl14* gene have been made and three have been published. These three independent mouse lines have shown reproducible skewed genotype ratios at weaning age compared to expected Mendelian ratios: crossing heterozygous mice produced only 2-12% *Cxcl14*^−/−^ mice instead of 25%; crossing heterozygous with homozygous mice only generated about 30% *Cxcl14*^−/−^ mice instead of the expected 50% [2–4]. It is still incompletely clear if reduced numbers of *Cxcl14*^−/−^ mice at weaning age is due to embryonic or perinatal mortality. CXCL14 appears important for the accumulation of uterine NK cells during pregnancy measured at embryonic day E13.5 [5]. Unfortunately, genotype ratios were not reported in that study. Data mentioned (but not shown) suggest perinatal death rather than embryonic mortality [3]. One cause of perinatal death may be the reduced antimicrobial activity normally provided by CXCL14 against opportunistic microbes. Exposure to bacteria during or shortly after birth may lead to sepsis and rapid death. Another cause of perinatal mortality of *Cxcl14*^−/−^ mice could be linked to reduced food intake and impaired adaptation abilities to a new environment as observed in adult *Cxcl14*^−/−^ mice [4]. This may then lead to out-competition by fitter wild-type or heterozygous littermates. However, the high mortality of newborn mice from homozygous mating also suggests intrinsic defects regardless of competition. Low birth weights in human infants and calorie-restricted pregnancies in macaques are associated with higher CXCL14 expression and methylation of *CXCL14* in umbilical cord blood and mesenchymal stem cells [6]. While CXCL14 expression and epigenetic changes may be biomarkers of an unfavorable environment during pregnancy, the consequences of epigenetic changes in CXCL14 are unknown. Finally, a third possibility for the abnormally high perinatal mortality of *Cxcl14*^−/−^ mice is that the targeting of *Cxcl14* affects the expression levels of a neighboring gene. *Cxcl14* is located 45kb upstream of *Neurog1* encoding Neurogenin 1, an important transcription factor regulating sensory neuron development. Incidentally, *Neurog1*^−/−^ mice die perinatally with 100% penetrance [7]. In conclusion, CXCL14 protein is an atypical, orphan chemokine that fulfills multiple functions in cellular recruitment, metabolism and microbial control. Further work is needed to determine which of these (or other) functions determine a favorable outcome in newborns.
